# Influencing factors of anxiety and depression of discharged COVID-19 patients in Wuhan, China

**DOI:** 10.1371/journal.pone.0276608

**Published:** 2022-11-16

**Authors:** Zhenwei Dai, Weijun Xiao, Hao Wang, Yijin Wu, Yiman Huang, Mingyu Si, Jiaqi Fu, Xu Chen, Mengmeng Jia, Zhiwei Leng, Dan Cui, Liming Dong, Winnie W. S. Mak, Xiaoyou Su

**Affiliations:** 1 School of Population Medicine and Public Health, Chinese Academy of Medical Sciences & Peking Union Medical College, Beijing, China; 2 National Clinical Research Center for Respiratory Diseases, China-Japan Friendship Hospital, Beijing, China; 3 The 2nd Affiliated Hospital of Harbin Medical University, Harbin Medical University, Harbin, China; 4 Diversity and Well-Being Laboratory, Department of Psychology, The Chinese University of Hong Kong, Shatin, New Territories, Hong Kong, China; Universidad Privada San Juan Bautista, PERU

## Abstract

**Objectives:**

This study is intended to assess the prevalence of depression and anxiety in individuals who had recovered from COVID-19 and been discharged from hospital (RD hereafter) in Wuhan, China, and to explore the factors associated with these mental disorders.

**Methods:**

Participants of this study were the RD who were infected at the beginning of the outbreak from 13 communities in Jianghan District of Wuhan City, Hubei Province, China by convenience sampling in mid-2021. The Generalized Anxiety Disorder Questionnaire, the Patient Health Questionnaire, the Short Version of COVID-19 Stigma Scale, the Peace of Mind Scale, the Resilience Style Questionnaire, and the Perceived Social Support Questionnaire were used to collect relevant information of the participants. Descriptive analyses, Pearson correlation analysis, and logistic regression analysis were used to describe and analyze the data and to examine the factors associated with the mental health status of this population.

**Results:**

In total, we recruited 1601 participants from 3059 COVID-19 patients, and 1541 participants completed the questionnaire survey, with a response rate of 96.25%. Finally, 1297 participants met the inclusion and exclusion criteria in this study, of whom 28.8% and 37.9% reported mild to severe levels of anxiety and depression symptoms. Perceived better mental health status during hospitalization, higher frequency of alcohol use per week, peace of mind, higher education level, and resilience were negatively associated with anxiety, while stigma and history of psychological or emotional counseling before infection was positively associated with anxiety. More severe clinical classification of COVID-19 and stigma (AOR = 1.057, P<0.001) were both positively associated with depression, while perceived better mental health status during hospitalization (AOR = 0.564, P<0.001), higher frequency of alcohol use per week (AOR = 0.462, P = 0.004), peace of mind (AOR = 0.857, P<0.001), and social support (AOR = 0.972, P = 0.034) were negatively associated with depression.

**Conclusions:**

Tailored interventions on reducing stigma, enhancing mindfulness and social support should be taken into account to alleviate anxiety and depression among RD.

## Introduction

As a major public health threat in the 21st century, the coronavirus disease 2019 (COVID-19) pandemic has caused tremendous mental health impact worldwide, including among those ever infected with the virus at the beginning of the outbreak [1, 2]. While most of the hospitalized COVID-19 patients had recovered and been discharged from hospital after effective treatments, the rehabilitation of both physical and mental health of RD has become a growing concern recently [3].

The uncertainties affected by the mass lockdowns and economic recession, and the lingering ‘long covid’ illness (subsequent sequela in the aftermath of COVID-19 infection, both mental and physical), along with the stigma they confronted from the surroundings, may cause mental disorders and even suicide among the RD population [4]. The National Institutes of Health (NIH) describes “long-covid” as sequelae that extend beyond 4 weeks after initial infection [5]. Evidence suggested that COVID-19 had led to unprecedented hazards to the mental health of those who used to be infected by COVID-19, since COVID-19 could infect brain or trigger immune responses that had additional adverse effects on brain functioning as well as affect psychological outcomes [6–10]. For severe or critical COVID-19 patients, the experience of long stay in intensive care unit (ICU), severe symptoms like respiratory failure, pains caused by clinical intervention, clinical sequelae associated with COVID-19, and fear of death could contribute to their traumatic stress during and after their hospitalization [11–16]. For patients with mild or moderate symptoms, multiple factors like fear of recurrence, clinical sequelae, and stigma, could also lead to negative psychological consequences after discharge [14–16]. Previous studies identified that anxiety and depression were common mental health problems that occurred among RD. A cross-sectional survey conducted after the outbreak in Wuhan, China in January, 2020 showed that the prevalence of anxiety and depression among COVID-19 patients were 56.3% and 39.3%, respectively [17]. Another survey in Wuhan in April, 2020 demonstrated that 42.7% of COVID-19 survivors had symptoms of anxiety, and 65.7% depression [18].

Reports suggested that psychological problems among RD have multiple risk and protective factors. Discrimination and stigma due to the experience of infection were common among RD [19, 20]. A survey conducted in Korea showed that stigma caused by COVID-19 infection could contribute to patients’ negative psychological symptoms [21]. Additionally, a previous study suggested that individuals with a peaceful mind tended to have better self-regulation capacity, especially affect-regulation capacity, and could upregulate positive affect as a way to cope with negative experiences [22]. In this case, peace of mind could be a positive factor associated with mental health of RD. Resilience, as a dynamic system to cope with adverse events and stress, could also serve as a positive indicator of mental health in various populations during the pandemic [23]. Moreover, social support was shown to be a significant factor in alleviating psychological distress among COVID-19 patients and other population [24].

Since psychological distress is common among RD, it is of importance to identify the psychological symptoms and the associated factors among this population [25]. However, relevant evidence in this area has not been well characterized, especially among the RD in China. Based on the protocol we developed previously [3], the present cross-sectional study aims to investigate the prevalence of anxiety and depression in RD among participants who were infected at the beginning of the outbreak in Wuhan, China, and to examine their possible influencing factors, which is critical in psychological intervention of COVID-19 patients.

## Materials and methods

### Sampling and participants

The cross-sectional study was carried out among former COVID-19 patients in Jianghan District (Wuhan, China) from June 10 to July 25, 2021. According to the electronic medical records of the Health Bureau of Jianghan District and inclusion criteria, a total of 3059 COVID-19 patients were eligible for the study and they were infected with the original SARS-Cov-2 strain and were diagnosed between December 10, 2019 and April 20, 2020. Among them, 1601 COVID-19 survivors were invited for a questionnaire survey on their mental health status when they were receiving clinical re-examination, and 1541 completed the survey and were included in the study, with a response rate of 96.25%. All investigators and support staff in this study were trained based on the same protocol and were required to have an educational background in medicine or public health. Those who had a history of COVID-19 infection, and ever have been hospitalized and discharged were invited to complete an online structured questionnaire from June to July, 2021. Digital informed consent was obtained from all individuals to ensure their voluntary participation. Self-administered electronic questionnaires and digital consent were sent to patients through Redcap, an online survey platform that is used for delivering online questionnaire. After reading the informed consent form and clicking the “I agree to participate in this study” button on the first page, participants could continue to fill in the electronic questionnaire. Ethics approval for the questionnaire survey was obtained from the Ethics Review Committee of the Institute of Pathogen Biology, Chinese Academy of Medical Sciences, Beijing, China (IPB-2020-22). The participants had to meet the following criteria: 1) over 18 years old; 2) had a history of hospitalization due to COVID-19 infection; 3) proficiency in Chinese; 4) able to independently cooperate with researchers to complete various scale assessments; 5) had a mobile communication equipment such as a mobile phone, and a WeChat account; 6) able to access the Internet with mobile equipment at any time; 7) had not received interventions for mental disorders within 1 month prior to enrollment or currently in the study. Those who met any of the below criteria were excluded: 1) had serious cognitive impairment; 2) had serious heart, brain, lung, kidney, liver, and other medical diseases or tumors; 3) difficult to cooperate with the questionnaire study. In total, 1297 participants were included in the final analysis based on the criterion above.

### Measures

#### Demographic characteristics

Demographic characteristics, including age (“≤60” or >”60”), gender (“Man” or “Woman”), marital status(“Married” or “Unmarried/divorced /widowed”), income for 2020 (“<60000 yuan” or “≥60000 yuan”), dwelling state ("Living alone” or “Living together”), education level (“Senior high school or below” or “Above senior high school”); and items on COVID-19 infection, such as family members’ infection, friends’ infection, length of hospital stay (“≤20 days” or “>20 days”), experience at ICU, clinical classification of COVID-19 (“Asymptomatic”, “Mild”, “Moderate”, and “Critically severe”), perceived mental health status during hospitalization (“Poor”, “Moderate”, or “Good”), etc. were asked.

#### Anxiety

The 7-item Generalized Anxiety Disorder Questionnaire (GAD-7) consists of 7 items that are rated on a 4-point Likert scaled from 0–3. It was developed for measuring the severity of generalized anxiety symptoms during the past two weeks [26]. The scores of the instrument range from 0 to 21. A cutoff score of ≥5 is recommended for considering significant anxiety symptoms, and scores between 5 and 9, 10 and 14, and 15 and higher represent mild, moderate, and severe anxiety symptoms respectively. This instrument has demonstrated to be reliable and valid among the Chinese population [27, 28]. In this study, the Cronbach’s alpha of the instrument was 0.951.

#### Depression

The 9-item Patient Health Questionnaire (PHQ-9) is a 9-item questionnaire that is used for screening and monitoring depression of varying degrees of severity during the past two weeks [29]. The items of the PHQ-9 are rated on a 4-point Likert scaled ranging from 0 to 3. The total score is utilized to assess the degree of depression of participants, with scores of ≥5 indicating depression, and scores of 5–9 mild depression; 10–14 moderate depression; 15–19 moderately severe depression; and scores of ≥20 severe depression. This instrument has been validated among various Chinese populations [30, 31]. In this study, the Cronbach’s alpha of the instrument was 0.914.

#### Stigma

The Short Version of COVID-19 Stigma Scale is a 12-item scale that is employed for evaluating the perceived stigma of patients of COVID-19 during the past two weeks [32]. Each item is scored on a Likert scale of 1–4. Higher total scores indicate greater stigmatization. In this study, the Cronbach’s alpha of the instrument was 0.936.

#### Peace of mind

The Peace of Mind Scale (PoM) comprises a total of 7 items rated on a 5-point scale ranging from 1 (“not at all”) to 5 (“all of the time”) and is used for measuring the peace of mind during the past two weeks [33]. Higher total scores indicate a more peaceful mind. In this study, the Cronbach’s alpha of the instrument was 0.874.

#### Resilience

The Resilience Style Questionnaire (RSQ) consists of 16 items that are rated on a 5-point Likert scaled from 1 to 5. It is used to measure the level of individual’s resilience during the past two weeks [34]. Higher total scores of the 16 items indicate a greater ability to recover from negative events. This instrument was developed and validated among the Chinese population [35, 36]. In this study, the Cronbach’s alpha of the instrument was 0.975.

#### Perceived social support

The level of perceived social support of the participants was measured by two items including emotional support and material support during the past two weeks [37]. The items were: 1) “How much support can you obtain from family/friends/colleagues when you need to talk or to obtain emotional support?” and 2) “How much support can you obtain from family/friends/colleagues when you need material support (e.g., financial help)?” and each item was 11-point Likert scaled from 0–10. In this study, the Cronbach’s alpha of the instrument was 0.819.

### Statistical analysis

Descriptive analyses were performed to describe the participants’ demographic and clinical characteristics, the prevalence of psychological problems, and potential influencing factors. Pearson correlation analysis was employed to estimate the association between psychological variables. Univariate logistic regression analysis began with the full set of demographics to evaluate their associations with anxiety and depression. As suggested by previous research, the type I error rate could be relaxed in univariate logistic regression to avoid omitting possible influencing factors. Hence, statistically significant variables at the level of p≤0.10 in the univariate analysis as well as stigma, peace of mind, resilience, and social support were further entered into the multivariate logistic regression analysis [38]. In the multivariate logistic regression analysis, statistically significant variables in the univariate analysis, stigma, peace of mind, resilience, and social support were the independent variables; depression and anxiety were the dependent variables. Adjusted odds ratio (AOR) and the corresponding 95% confidence intervals (95% CI) were calculated to assess the results of the regression model. SAS9.4 was utilized to conduct all the analysis with level of significance determined at a 0.05 p-value.

## Results

### Demographic characteristics

Among the 1297 participants, 47.3% aged above 60 years old; 56.6% were men; 85.2% were married; 62.1% had an annual family income of less than RMB 60000 in 2020; 12.2% lived alone; 29.0% were at an educational level of undergraduate or above; 46.5% had other family members infected with COVID-19; 29.1% had friends or relatives infected with COVID-19; 47.1% were hospitalized for over 20 days; 3.6% used to be treated at intensive care unit (ICU); 13.7% were in critical condition after infection; 56.7% perceived good psychological status during infection; 9.9% had a history of psychological or emotional counseling before infection; 12.4% were current smoker; 9.0% drank more than 2 times a week. The results are displayed in Table 1.

**Table 1:** Demographic and clinical characteristics of participants.

Variable	N	%
**Age (years)**		
≤60	683	52.70
>60	614	47.30
**Gender**		
Woman	734	43.40
Man	563	56.60
**Marital status**		
Married	1105	85.20
Unmarried/divorced /widowed	192	14.80
**Income for 2020 (CNY)**		
<60000	805	62.10
≧60000	492	37.90
**Dwelling state**		
Living alone	158	12.20
Living together	1139	87.80
**Education level**		
Senior high school or below	921	71.00
Above senior high school	376	29.00
**Family members’ infection**		
Confirmed infection	603	46.50
No infection	694	53.50
**Friends’ infection**		
Confirmed infection	378	29.10
No infection	909	70.10
**Length of hospital stay, days**		
≤20	686	52.90
>20	611	47.10
**Experience at ICU**		
Yes	47	3.60
No	1250	96.40
**Clinical classification of COVID-19 patients**		
Asymptomatic	60	4.60
Mild	927	71.50
Moderate	132	10.20
Critically severe	178	13.70
**Perceived mental health status during hospitalization**		
Poor	314	24.20
Moderate	247	19.00
Good	736	56.70
**Having received psychological or emotional counseling before infection**		
Yes	128	9.90
No	1169	90.10
**Current smoker**		
Yes	161	12.40
No	1136	87.60
**Frequency of alcohol use per week**		
< 2	1180	91.00
≧2	117	9.00

### Prevalence of anxiety and depression of RD

In this study, 28.8% and 37.9% of the participants reported mild to severe levels of anxiety and depression symptoms according to the cut-off values of GAD-7 and PHQ-9, as shown in Table 2.

**Table 2:** Mental health status of enrolled COVID-19 survivors.

Variable	n	%	Mean (SD)	Range
**Stigma** (**Covid-19 Stigma Scale**)				
Total score			28.04(7.33)	12–48
**Peace of mind** (**Peace of Mind Scale**)				
Total score			24.70(5.99)	7–35
**Resilience (RSQ)**				
Total score			56.82(14.04)	16–80
**Social support**				
Total score			14.25(5.18)	0–20
**Anxiety**				
No	923	71.2		
Mild	299	23.1		
Moderate	46	3.5		
Severe	29	2.2		
**Depression**				
No	805	62.1		
Mild	315	24.3		
Moderate	110	8.5		
Moderate severe	48	3.7		
Severe	19	1.5		

### Associations of psychological symptoms in the participants

The associations of anxiety, depression, stigma, resilience, peace of mind, and social support among the RD were all statistically significant at a 0.01 p-value, where anxiety and depression were all positively associated with stigma, and negatively associated with resilience, peace of mind, and social support. The results are illustrated in Table 3.

**Table 3:** Correlation matrix of mental health status in enrolled COVID-19 survivors with Pearson correlation analysis.

	Depression	Anxiety	Stigma	Psychological resilience	Peace of mind	Social support
Depression	1					
Anxiety	0.824**	1				
Stigma	0.323**	0.330**	1			
Resilience	-0.213**	-0.206**	-0.125**	1		
Peace of mind	-0.190**	-0.215**	-0.063**	0.542**	1	
Social support	-0.256**	-0.222**	-0.181**	0.223**	0.207**	1

Note.

**: P < 0.01.

### Variables associated with anxiety and depression of RD

The factors associated with anxiety and depression of the participants are presented in Tables 4 and 5. The univariate logistic regression analysis revealed that woman, experience at ICU, more severe clinical classification of COVID-19 infection, and history of psychological or emotional counseling before infection were positively associated with anxiety and depression, while higher family income for 2020, higher education level, perceived better mental health status during hospitalization, and higher frequency of alcohol use per week were negatively associated with the prevalence of anxiety and depression. These factors were later employed in multivariate logistic regression with stigma, peace of mind, resilience, and social support, to identify the potential influencing factors of anxiety and depression among RD.

**Table 4:** Univariate statistical analysis of adverse psychological outcomes in enrolled COVID-19 survivors.

	Anxiety		Depression	
	OR (95%CI)	P value	OR (95%CI)	P value
**Age(years)**				
≤60	1		1	
>60	0.941(0.739–1.197)	0.619	1.041(0.832–1.304)	0.732
**Gender**				
Man	1		1	
Woman	1.555 (1.214–1.993)	<0.001	1.489 (1.184–1.873)	0.001
**Marital status**				
Unmarried/divorced /widowed	1		1	
Married	0.849 (0.610–1.181)	0.331	0.832 (0.609–1.137)	0.832
**Family income for 2020 (CNY)**				
<60000	1		1	
≧60000	0.632 (0.489–0.817)	<0.001	0.687 (0.543–0.869)	0.002
**Dwelling state**				
Living alone	1		1	
Living with other people	0.888 (0.619–1.274)	0.519	1.029 (0.730–1.451)	0.870
**Education level**				
Senior high school or below	1		1	
Above senior high school	0.589 (0.444–0.780)	<0.001	0.739 (0.575–0.952)	0.019
**Family members’ infection**				
No infection	1		1	
Confirmed infection	1.201 (0.944–1.527)	0.137	1.017 (0.812–1.273)	0.885
**Friends’ infection**				
No infection	1		1	
Confirmed infection	1.0978 (0.829–1.402)	0.576	1.108 (0.867–1.417)	0.413
**Length of hospital stay(days)**				
≤20	1		1	
>20	1.043(0.820–1.327)	0.730	1.174(0.938–1.470)	0.161
**Experience at ICU**				
No	1		1	
Yes	2.245 (1.249–4.034)	0.007	2.083 (1.159–3.744)	0.014
**Clinical classification of COVID-19 patients**				
Asymptomatic	1		1	
Mild	1.402 (0.746–2.634)	0.294	1.542 (0.857–2.775)	0.149
Moderate	1.870 (0.918–3.811)	0.085	2.363 (1.213–4.602)	0.011
Critical severe	1.703 (0.854–3.396)	0.131	2.349 (1.234–4.471)	0.009
**Perceived mental health status during hospitalization**				
Poor	1		1	
Moderate	0.659 (0.467–0.928)	0.017	0.669 (0.479–0.935)	0.019
Good	0.297 (0.223–0.396)	<0.001	0.345 (0.263–0.453)	<0.001
**Having received psychological or emotional counseling**				
No	1		1	
Yes	2.008 (1.383–2.915)	<0.001	1.793 (1.243–2.586)	0.002
**Current smoker**				
No	1		1	
Yes	0.794 (0.543–1.160)	0.233	0.830 (0.587–1.174)	0.292
**Frequency of alcohol use per week**				
< 2	1		1	
≧2	0.512 (0.314–0.835)	0.007	0.485 (0.313–0.754)	0.001

**Table 5:** Multivariate statistical analysis of adverse psychological outcomes in enrolled COVID-19 survivors.

	Anxiety		Depression	
	AOR (95%CI)	P value	AOR (95%CI)	P value
**Gender**				
Man	1		1	
Woman	1.171 (0.858–1.600)	0.320	1.204 (0.908–1.597)	0.198
**Family income for 2020 (CNY)**				
<60000	1		1	
≧60000	0.826 (0.595–1.148)	0.255	0.825 (0.614–1.110)	0.204
**Education level**				
Senior high school or below	1		1	
Above senior high school	0.727 (0.505–1.048)	0.088	0.992 (0.715–1.377)	0.964
**Experience at ICU**				
No	1		1	
Yes	1.627 (0.754–3.513)	0.215	1.310 (0.626–2.744)	0.474
**Clinical classification of COVID-19 patients**				
Asymptomatic	1		1	
Mild	1.702 (0.809–3.583)	0.161	1.818 (0.924–3.579)	0.084
Moderate	1.714 (0.738–3.981)	0.210	2.318 (1.073–5.006)	0.032
Critical severe	1.287 (0.560–2.960)	0.552	2.235 (1.047–4.770)	0.038
**Perceived mental health status during hospitalization**				
Poor	1		1	
Moderate	0.944 (0.630–1.416)	0.782	0.989 (0.672–1.456)	0.956
Good	0.441 (0.312–0.625)	<0.001	0.564 (0.409–0.778)	<0.001
**Received psychological or emotional counseling**				
No	1		1	
Yes	1.485 (0.941–2.346)	0.090	1.269 (0.821–1.964)	0.284
**Frequency of alcohol use per week**				
< 2	1		1	
≧2	0.472 (0.264–0.843)	0.011	0.462 (0.275–0.778)	0.004
**Stigma**	1.068(1.045–1.090)	<0.001	1.057 (1.037–1.078)	<0.001
**Peace of mind**	0.826(0.798–0.855)	<0.001	0.857 (0.831–0.883)	<0.001
**Resilience**	0.989(0.977–1.001)	0.074	1.007 (0.995–1.019)	0.236
**Social support**	0.989(0.961–1.018)	0.446	0.972 (0.947–0.998)	0.034

In multivariate analysis, we conducted the diagnostic evaluation of the model, the samples were all independent and the variables showed no multicollinearity. The multivariate analysis revealed that perceived better mental health status during hospitalization (AOR = 0.441, P<0.001), higher frequency of alcohol use per week (AOR = 0.472, P = 0.011), and peace of mind (AOR = 0.826, P<0.001) were negatively associated with anxiety, and a non-significant trend was observed in negative associations between anxiety and higher education level (AOR = 0.727, P = 0.088), and resilience (AOR = 0.989, P = 0.074). While stigma (AOR = 1.068, P<0.001) was positively associated with anxiety, and non-significant trends were observed in positive associations between anxiety and history of psychological or emotional counseling before infection (AOR = 1.485, P = 0.090). Moreover, more severe clinical classification of COVID-19 (AOR = 1.714/1.287, P = 0.032/0.038) and stigma (AOR = 1.057, P<0.001) were both positively associated with depression, while perceived better mental health status during hospitalization (AOR = 0.564, P<0.001), higher frequency of alcohol use per week (AOR = 0.462, P = 0.004), peace of mind (AOR = 0.857, P<0.001), and social support (AOR = 0.972, P = 0.034) were negatively associated with depression.

## Discussion

Mental disorders among RD are associated with their prognosis and may lead to more severe psychological sequelae without effective control [39]. Hence, this study aimed to investigate the conditions of anxiety and depression and examine their influencing factors among RD in China from an epidemiological perspective, which could help to understand the mental health problems in RD more comprehensively, and provide a reference for the development of related policy [40] In this study, the prevalence of anxiety and depression among participants were 28.8% and 37.9% respectively, both lower than the results of two surveys conducted among COVID-19 patients in January and April 2020 in Wuhan, China [17, 18]. Despite the steady overall improvement of mental health in RD over time, the current study showed that their mental health status was still worse than those who had never been infected [41–43]. In this case, psychological distress caused by COVID-19 infection should not be ignored in the time of long covid [44].

Based on the current study sample, we found that COVID-19 related stigma was positively associated with the occurrence of anxiety and depression among RD. Stigma has become a public health challenge during the COVID-19 pandemic and might cause severe mental illness in RD. Infected individuals who perceived being stigmatized could report guilt, self-isolation, self-depreciation, the experience of social exclusion, stereotyping, and insulation, hence they often suffer from psychiatric comorbidities, such as PTSD, anxiety, depression, and even suicidal ideation [45, 46]. Stigma due to fear of being re-infected was still common among RD in China, and the health authorities should enhance the appeals to stop stigmatizing RD, and improve public awareness and knowledge on the dynamics and modes of transmission of COVID-19 [46]. Moreover, social media were suggested to contribute to eliminating stigma by challenging stereotyping and stigmatization, spreading knowledge on COVID-19 to a wider audience, stopping rumors, and dispelling misinformation and conspiracy theories on COVID-19 [47].

In addition, the results of this study suggested that RD who had more severe clinical classification during hospitalization were more likely to develop depression. This finding is similar to the result of a multi-national cohort study in RD across six countries [48]. Literature also revealed RD who used to be bedridden for a week or longer were more intended to have obvious long-term anxiety and depression symptoms, while a mild course of acute illness of RD, such as fewer bedridden, was associated with their lower risk of mental disorders [48]. The possibility of infecting others, the unpredictable prognosis of COVID-19, and continued sequelae of COVID-19 may cause worry and helplessness among RD with more severe symptoms during hospitalization, thus leading to depression [48]. Accordingly, more attention should be paid to the mental health morbidities among RD with more severe symptomology during hospitalization, especially those who used to be bedridden for a week or longer [48].

By contrast, we found that perceived better mental health status during hospitalization and social support were protective factors against mental disorders among RD. Consistent with previous researches, RD who could perceive good mental health status usually viewed negative life events more optimistically, and could adapt themselves better to new lives after discharge, thus reducing the possibility of having psychological problems [49, 50]. Higher level of social support may help RD perceive the respect and care from their family, friends, and others more easily, hence, their depressive symptoms could be buffered when encountering negative events [24, 51, 52]. Government could consider enhancing social support for RD by providing more emotional and material support for them [53]. Additionally, health authorities could consider encouraging RD to actively participate in social activities and improve peer support, to alleviate their self-discrimination and negative emotions, thus preventing the occurrence of depression [54–56]. Furthermore, relative social media should consider appealing general population to support RD, to help RD perceive the support from their surroundings more easily and their depressive disorders could be reduced consequently [57, 58].

Additionally, findings of this study indicated that peace of mind and resilience were negatively related to mental disorders among RD. Previous research has suggested that peace of mind is associated with anxiety and depression [22]. Individuals with a higher level of peaceful mind were more inclined to experience inner peace and harmony, and could achieve a balance between positive and negative emotions, thus reducing the occurrence of psychological problems [33]. Despite a non-significant trend in this study, higher levels of psychological resilience could enable RD to take positive coping styles and mitigate the experience of anxious disorders [59]. Resilience is a dynamic procedure to help individuals positively adapt to adverse contexts [60, 61]. Some literature also implied that psychological resilience might be a translational endpoint in the treatment of mental disorders [62, 63]. Evidence suggested that mindfulness practice could improve individuals’ peace of mind [64]. Mindfulness practice centers on cultivating intentional awareness in unfolding present-moment experiences to achieve inner peace, which is associated with peace of mind in nature [65, 66]. Peace of mind was listed as an outcome in our mindfulness training protocol for RD in Wuhan [3]. As suggested by the Wuhan protocol, online mindfulness intervention via mobile phone could be more convenient and effective compared to face-to-face mindfulness practice. Since mindfulness intervention has been commonly used among various populations and proved effective to improve mental health, the health authorities could consider employing online mindfulness practice among RD to increase their peace of mind, thus reducing the occurrence of anxiety, and depression symptoms [67, 68]. Similar to peace of mind, the effect of mindfulness intervention on improving psychological resilience was obvious [69]. Therefore, relative health authorities could consider implementing mindfulness intervention in RD to improve their psychological resilience and peace of mind simultaneously. On the other hand, other interventions on psychological resilience such as cognitive-behavioral therapy, acceptance and commitment therapy, and problem-solving therapy could also be taken into account [70].

Although previous systematic reviews indicated substantial harm of alcohol use on health, the current study found alcohol use could protect RD from both anxiety and depression [71]. A cross-sectional study indicated that low to moderate alcohol use could alleviate depressive symptomatology among the elderly in the US [72]. A cohort study also suggested that low to moderate alcohol drinking was associated with better cognitive functions among middle-aged and elderly adults in the US [73]. However, while certain amount of alcohol use might improve individual’s mental health to some extent, this did not apply to binge drinking, since binge drinking could harm mental health in the long term [74–76]. Moreover, alcohol consumption may increase pro-inflammatory markers and the mortality risk due to COVID-19-associated complications in RD [77]. Hence, although the present study suggested possible benefits of alcohol, public health agencies should remind RD of the long-term effects of drinking on mental health, and the importance of avoiding binge drinking.

Moreover, the findings of this study indicated that RD with a history of psychological or emotional counseling before infection might be at higher risk of anxiety. Evidence suggested that individuals with a history of psychological counseling might have experienced psychological distress before, and the distress was more likely to reoccur due to COVID-19 infection among RD [78, 79]. This finding suggested that more attention should be paid to the mental health of RD with a history of psychological or emotional counseling, and it is essential to prevent the reoccurrence of psychological problems among this population.

Overall, findings of this study suggested that the government, health authorities, as well as social media could consider alleviating mental disorders among RD from several dimensions, such as mobilizing society to stop stigmatizing, providing more support for RD, and implementing psychological interventions in RD.

This study has several limitations. First, a drawback of the cross-sectional study is that we can’t make any statements regarding causality. As a consequence, findings on risk factors of adverse psychological outcomes should be accepted with caution. Second, our study was conducted more than 18 months after discharge of COVID-19 patients, which may lead to recall bias. Third, convenience sampling may decrease the representativeness of the population, and cause biased calculation of confidence interval in the logistic regression analysis. Fourth, the results may be biased due to various access to technology, connectivity, and skills to use mobile communication equipment. Fifth, we did not consider the heterogeneity between 13 communities of the jianghan district, which may cause bias. Sixth, in the categorization of some demographic variables, such as income and length of hospital stay, we set the cutoff point based on the data characteristics, which may be a little subjective. Seventh, in the evaluation of alcohol use, we only consider the drinking frequency without alcohol volumes, which may not accurate enough to assess alcohol use. Eighth, we recruited participants who had not received interventions for mental disorders within 1 month prior to enrollment without considering the bias caused by having received any type of psychological intervention two or more months prior to the survey. In addition, our study didn’t collect mental health information about patients who died of COVID-19, which may underestimate the rate of mental health problems in discharged patients infected with COVID-19.

## Conclusion

The prevalence of anxiety and depression in people who had recovered from COVID-19 and had been discharged from hospital in Wuhan, China were 28.8% and 37.9% in this study. We found that stigma caused by COVID-19 infection was positively associated with mental disorders, while social support, peace of mind, psychological resilience, and alcohol use were negatively associated with mental disorders like anxiety and depression in RD. More attention should be paid to RD with a history of psychological or emotional counseling before infection. Tailored interventions regarding reducing stigma, enhancing mindfulness level and social support should be taken into account to alleviate anxiety and depression among RD. Further research could focus on prospective studies or long-term follow-up to examine the causal relations between the factors identified in this study.

## Supporting information

S1 Data (XLSX)
